# Prognostic impact of fecal pH in critically ill patients

**DOI:** 10.1186/cc11413

**Published:** 2012-07-10

**Authors:** Akinori Osuka, Kentaro Shimizu, Hiroshi Ogura, Osamu Tasaki, Toshimitsu Hamasaki, Takashi Asahara, Koji Nomoto, Masami Morotomi, Yasuyuki Kuwagata, Takeshi Shimazu

**Affiliations:** 1 Department of Traumatology and Acute Critical Medicine, Osaka University Graduate School of Medicine, 2-15 Yamadaoka, Suita, Osaka, 565-0871, Japan; 2 Department of Clinical Quality Management, Osaka University Hospital, 2-15 Yamadaoka, Suita, Osaka, 565-0871, Japan; 3 Department of Biomedical Statistics, Osaka University Graduate School of Medicine, 2-15 Yamadaoka, Suita, Osaka, 565-0871, Japan; 4 Yakult Central Institute for Microbiological Research, 1796 Yaho, Kunitachi, Tokyo, 186-8650, Japan

## Abstract

**Introduction:**

We have reported that altered gut flora is associated with septic complications and eventual death in critically ill patients with systemic inflammatory response syndrome. It is unclear how fecal pH affects these patients. We sought to determine whether fecal pH can be used as an assessment tool for the clinical course of critically ill patients.

**Methods:**

Four hundred ninety-one fecal samples were collected from 138 patients who were admitted to the Department of Traumatology and Acute Critical Medicine, Osaka University Graduate School of Medicine, Japan. These patients were treated in the intensive care unit for more than 2 days. Fecal pH, fecal organic acids, and fecal bacteria counts were measured and compared by survived group and nonsurvived group, or nonbacteremia group and bacteremia group. Logistic regression was used to estimate relations between fecal pH, age, sex, or APACHE II score and mortality, and incidence of bacteremia. Differences in fecal organic acids or fecal bacteria counts among acidic, neutral, and alkaline feces were analyzed.

**Results:**

The increase of fecal pH 6.6 was significantly associated with the increased mortality (odds ratio, 2.46; 95% confidence interval, 1.25 to 4.82) or incidence of bacteremia (3.25; 1.67 to 6.30). Total organic acid was increased in acidic feces and decreased in alkaline feces. Lactic acid, succinic acid, and formic acid were the main contributors to acidity in acidic feces. In alkaline feces, acetic acid was significantly decreased. Propionic acid was markedly decreased in both acidic and alkaline feces compared with neutral feces. No differences were noted among the groups in bacterial counts.

**Conclusions:**

The data presented here demonstrate that the fecal pH range that extended beyond the normal range was associated with the clinical course and prognosis of critically ill patients.

## Introduction

The gut has been described as the "motor" of multiple organ failure syndrome (MODS) [1], and is now considered a crucial target organ after severe insults such as trauma and sepsis. The gut has an important role in promoting infectious complications and MODS. This is due to deteriorated intestinal epithelia, the immune system, and commensal bacteria [2].

Recently, we evaluated microflora and environmental changes in patients with severe systemic inflammatory response syndrome (SIRS). Analysis of fecal flora confirmed that patients with severe SIRS had significantly lower total anerobic bacteria counts (particularly *Bifidobacterium *and *Lactobacillus*) and higher *Staphylococcus *and *Pseudomonas *group counts than did healthy volunteers. Concentrations of total organic acids, particularly of beneficial short-chain fatty acids (SCFAs), such as acetic acid, propionic acid, and butyric acid in the feces, were significantly decreased in these patients [3]. We also found that altered gut flora was associated with gastrointestinal dysmotility [4].

In this study, we wished to determine the impact of fecal pH in critically ill patients. Little is known about the relation between fecal pH and critical illness. However, it has been demonstrated that gastrointestinal pH has a significant impact on the absorption of vitamins and electrolytes and the activity of digestive enzymes [5]. We have reported that fecal pH is markedly increased in patients with severe SIRS [3]. We therefore hypothesized that fecal pH beyond the normal range predicts the clinical course and prognosis of critically ill patients. The objective of this study was to clarify the significance of fecal pH as a marker for assessment of critically ill patients.

## Materials and methods

### Patients

One hundred thirty-eight patients were recruited from admissions to the Department of Traumatology and Acute Critical Medicine, Osaka University Graduate School of Medicine, Japan, who were treated in the intensive care unit for more than 2 days from January 1, 2003, to March 31, 2009. When infectious complications occurred, antibiotics were administered based on the underlying clinical symptoms and results of microbiologic cultures and Gram staining. Fecal samples were obtained serially after admission and analyzed.

This study was approved by the Institutional Review Board of Osaka University, and informed consent was obtained from the patients and/or their families.

### Measurements of fecal pH

The fecal pH was measured by direct insertion of stainless steel pH probes of an IQ150 pH meter system (IQ Scientific Instruments, Inc., Carlsbad, CA, USA) into the homogenized feces. A normal range was defined based on the readings of 14 healthy volunteers (6.6 ± 0.3; mean ± SD) [3,6,7]. To test our hypothesis, we used the furthest points from the normal average as representing each patient's fecal pH, which means the maximum points of the absolute value of pH 6.6.

### Determination of fecal organic acid concentrations

A portion of the feces was isolated, weighed, mixed with 0.15 *M *perchloric acid at a fourfold volume, and stored at 4°C for 12 hours. The mixture was centrifuged at 4°C at 12,000 rpm for 10 minutes. The supernatant was filtered with a 0.45-μm membrane filter (Millipore Japan Ltd., Tokyo, Japan) and sterilized. The sample was analyzed for organic acids with high-performance liquid chromatography, which was performed with a Waters system (Waters 432 Conductivity Detector; Waters Co., Milford, MA, USA) equipped with two columns (Shodex RSpack KC-811; Showa Denko Co. Ltd., Tokyo, Japan). The concentrations of organic acids were calculated by using external standards. The reproducibility and stability of these measurements have been shown previously [8].

### Fecal bacteriologic culture

Feces were collected in a test tube, which was maintained under anaerobic conditions in an atmosphere of 7% H_2 _and 5% CO_2 _in N_2_. The test tube was cooled in an icebox before culture. VL-G roll tube agar [9] supplemented with 0.2% cellobiose and 0.2% maltose (modified VL-G roll-tube agar) was used to determine total anaerobic counts. Different media were used for selective isolation of microorganisms: modified VL-G roll-tube agar supplemented with 80 μg/ml vancomycin and 1 μg/ml kanamycin for Bacteroidaceae; CW agar (Nikken Bio Medical Laboratory Inc., Kyoto, Japan) for lecithinase-positive *Clostridium*; MPN agar [10] for *Bifidobacterium*; COBA agar [11] for *Enterococcus*; LBS agar (Becton Dickinson and Company, Cockeysville, MD, USA) supplemented with 0.8% Laboratory Lemco powder (Oxoid Co. Ltd, Basingstoke, UK) for *Lactobacillus*; *Staphylococcus *medium no. 110 agar (Nissui Pharmaceutical Co., Ltd., Tokyo, Japan) for *Staphylococcus*; DHL agar (Nissui Pharmaceutical Co., Ltd.) for Enterobacteriaceae; NAC agar (Nissui Pharmaceutical Co., Ltd.) for *Pseudomonas*; and GS agar (Nissui Pharmaceutical Co., Ltd.) for *Candida*. CW agar and LBS agar were cultured anaerobically at 37°C for 72 hours. After incubation, colonies on plates were counted. Numbers of viable bacteria per gram of feces (wet weight) were calculated. All bacterial counts (colony-forming units (CFUs)/g of wet feces) were transformed to a logarithmic scale (log_10_CFU) for ease of statistical analysis. The lower limit of bacterial detection with this procedure was 1,000 CFU/g of feces for the obligate anaerobes, Bacteroidaceae, and *Bifidobacterium*, and 100 CFU/g of feces for other bacteria. The reproducibility and stability of these measurements was shown previously [8].

### Surveillance and definition of infections

We defined infectious complications as infections that occurred after the diagnosis of SIRS during the ICU stay. Body temperature was measured continuously. Surveillance cultures from urine, blood, and sputum were performed routinely for each patient. In cases of suspected infection, laboratory tests, chest radiographs, and computed tomography scanning were performed when necessary. Bacterial infection was diagnosed in accordance with the Centers for Disease Control Definitions [12]. Bacteremia was defined as at least one positive blood culture.

### Statistical analysis

The pH 6.6 in each patient was determined as the maximum value of pH 6.6 in serial fecal samples.

The effects of pH 6.6, age, sex, and APACHE II score on the incidence of bacteremia or mortality were analyzed with the multivariate logistic regression model, and the corresponding odds ratio with 95% confidence interval (CI) were calculated. The organic acids or bacterial counts were compared for the first time among four groups with a Tukey-type adjustment for multiple comparisons, in which the four groups were acidic (pH < 6.0), normal range (6.0 ≤ pH ≤ 7.2), alkaline (< 7.2 pH), healthy volunteers, where normal range was defined as within two standard deviations (±) of the mean. The results were summarized as mean values ± SEM. All tests were two-sided, and *P *< 0.05 was considered significant. The statistical analyses were performed with SAS for Windows 9.1.3 (SAS Institute, Cary, NC, USA).

## Results

**Figure 1:**
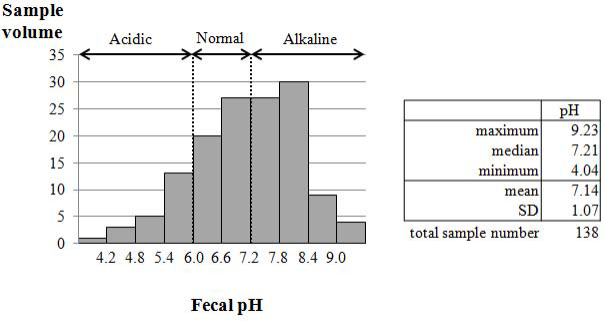
**Overview of fecal samples**. The histogram shows 138 sample data of pH. The table shows the distribution of the fecal pH.

We collected 491 fecal samples from 138 patients. Patient demographics are shown in Table 1. Figure 1 shows the distribution of fecal pH in each patient when pH 6.6 was the maximum. The pH ranged from 4.04 to 9.23, and the mean was 7.14, which was significantly increased (*P *< 0.01, Mann-Whitney test) compared with healthy volunteers (6.6 ± 0.3; mean ± SD). The patient characteristics, according to acidic, normal-range, and alkaline feces, are shown in Table 2. The incidence of bacteremia in the acidic or alkaline group was significantly higher than the normal range (*P *< 0.05 versus normal range; Pearson χ^2 ^test). All patients died of MODS. Analysis of blood cultures detected *Staphylococcus *species in 21 patients, *Enterococcus faecalis *in five patients, *Escherichia coli *in six patients, *Pseudomonas *in eight patients, and other bacteria in five patients.

**Table 1:** Patient characteristics with severe systemic inflammatory response syndrome (SIRS)

Characteristics	Value
Age (years)	59.0 ± 19.2^a^
Sex (male/female)	91/47
APACHE II	16.8 ± 7.4^a^
Origins of SIRS	
Sepsis	95
Pneumonia	31
Necrotizing fasciitis	20
Enteritis	6
Peritonitis	26
Catheter infection	7
Meningitis	2
Mediastinitis	3
Trauma	31
Head and neck	15
Thorax	4
Abdomen	8
Pelvis	4
Burn	12

^a^Mean ± standard deviation (SD); *n *= 138.

**Table 2:** Patient characteristics with acidic, normal-range, and alkaline feces

	Acidic (pH < 6.0, *n *= 22)	Normal range (pH ≤ 6.0 to ≤ 7.2; *n *= 47)	Alkaline (pH < 7.2; *n *= 69)
Age (years; mean ± SD)	60.6 ± 14.2	58.5 ± 19.8	58.9 ± 20.4

Sex (M/F)	15/7	35/12	41/28

APACHE II (mean ± SD)	17.6 ± 8.6	16.0 ± 7.9	17.1 ± 6.7

Bacteremia (%)	45.5^a^	12.8	31.9^a^

Mortality (%)	31.8	17.0	30.4

Origins of SIRS			

Infection	19	32	44

Trauma	2	10	19

Burn	1	5	6

Total	22	47	69

^a^*P *< 0.05 versus normal range, Pearson χ^2 ^test.

No significant differences in characteristics were found between groups. Table 3 shows the results of multivariate logistic regression analysis. The incidence of both bacteremia and mortality was associated with the increase of pH 6.6. The odds ratio indicates that when the pH level is increased or decreased by one, the incidence of bacteremia more than triples, and mortality more than doubles. Age was also significantly related with both mortality and the incidence of bacteremia. Sex and APACHE II score were not related to the clinical course. No correlation was seen between length of time in the ICU and the fecal pH (*P *= 0.61; Spearman nonparametric correlation test).

**Table 3:** Results of multivariate logistic regression analysis

							95% Confidence interval	
							
		Coeff (β)	SE (β)	Wald stat	*P *value	Odds ratio	Lower limit	Upper limit
Bacteremia	pH 6.6	1.18	0.3385	12.10	0.0005^a^	3.25	1.67	6.30
	Age	0.03	0.0136	3.98	0.0462^a^	1.03 (1.31 per 10)	1.00	1.06
	Sex (male/female)	0.79	0.4638	2.91	0.0879	2.21	0.89	5.48
	APACHE II	-0.03	0.0316	0.78	0.3782	0.97	0.91	1.04
Mortality	pH 6.6	0.90	0.3442	6.82	0.0090^a^	2.46	1.25	4.82
	Age	0.05	0.0157	8.90	0.0029^a^	1.05 (1.60 per 10)	1.02	1.08
	Sex (male/female)	0.76	0.4759	2.54	0.1113	2.13	0.84	5.42
	APACHE II	-0.01	0.0309	0.08	0.7769	0.99	0.93	1.05

Coeff (β), coefficient; SE (β), standard error of coefficient; ^a^*P *< 0.05.

**Figure 2:**
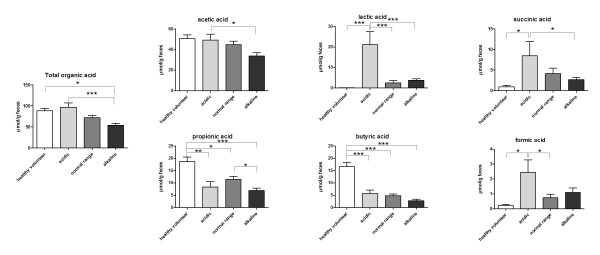
**Organic acid levels in acidic, normal range, and alkaline feces**. Feces from healthy volunteers were used as a baseline comparison control. Fecal samples were tested for organic acid levels, including detection of total organic acid, acetic acid, lactic acid, succinic acid, propionic acid, butyric acid, and formic acid. The data are plotted as mean ± SEM μmol/g of feces. *P < 0.05; **P < 0.01; and ***P < 0.001 by the Tukey multiple comparison test.

Figure 2 shows the organic acids levels in acidic, normal range, and alkaline feces. Lactic, succinic, and formic acids were the main contributors to acidity in acidic feces, although acetic acid was the predominant organic acid. In acidic feces, lactic (acidic versus normal range, 21.09 ± 6.45 versus 2.51 ± 1.16; mean ± standard error of the mean) and formic (acidic versus normal range, 2.46 ± 0.83 versus 0.74 ± 0.24) acids were significantly higher than normal-range feces, whereas no differences in acetic acid were noted between acidic and neutral feces (acidic versus normal range, 49.38 ± 5.65 versus 44.91 ± 3.35). Total organic acid was decreased in alkaline feces (alkaline versus normal range, 53.99 ± 4.61 versus 71.79 ± 5.33). Interestingly, propionic acid was markedly decreased in alkaline feces compared with normal-range feces (alkaline versus normal range, 6.91 ± 0.99 versus 11.45 ± 1.23). Furthermore, propionic and butyric acids were significantly decreased in all patient groups compared with healthy volunteers.

**Figure 3:**
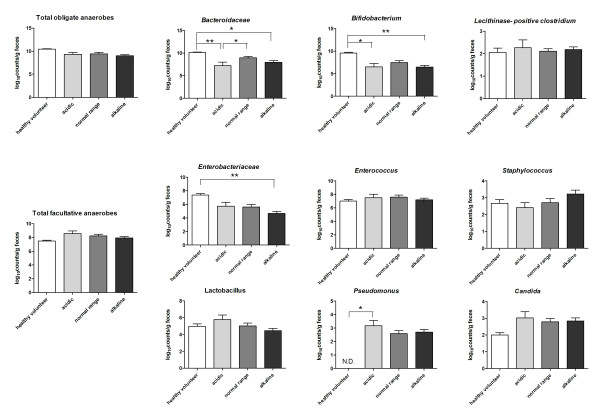
**Bacterial counts in acidic, normal-range, and alkaline feces**. Feces from healthy volunteers were used as a baseline comparison control. The data are plotted as mean ± SEM log_10 _CFU/g of feces. *P < 0.05; **P < 0.01; and ***P < 0.001 by the Tukey multiple comparison test. ND, not detected.

The bacterial counts are shown in Figure 3. No differences in total obligate anaerobes were noted between the groups. Only Bacteroidaceae were significantly decreased in acidic compared with normal range (acidic versus normal range, 7.24 ± 0.75 versus 8.99 ± 0.30). Compared with feces from healthy patients, Bacteroidaceae and *Bifidobacterium *sp. were significantly decreased in both acidic and alkaline feces. In contrast, only Enterobacteriaceae were decreased in alkaline feces among the facultative anaerobes analyses. *Pseudomonas *spp. were detected only in patient groups.

## Discussion

The gut is known to play an important role in critically ill patients. For example, shock induces gut hypoperfusion, leading to production of proinflammatory mediators, which can amplify the SIRS response [13]. Fink and colleagues [14-16] reported that epithelial tight junctions are compromised in critical illness, leading to increased permeability and persistent activation of systemic inflammation. Even social-disruption stress can increase the translocation of gastrointestinal microbiota to secondary lymphoid organs [17]. Our group previously demonstrated that altered gut flora are associated with septic complications and death in critically ill patients [3].

Although it has been shown that patients with severe SIRS have altered gut environments, still little is known about the relation between fecal pH and critical illness. To the best of our knowledge, the relation between fecal pH and SIRS was first described by our group in 2006 [3]. The present study was initiated to test the hypothesis that drastic changes in fecal pH in critical illness contribute to the development of bacteremia or death. Our findings presented here establish that fecal pH changes are associated with increased incidence of bacteremia and mortality.

Given the association between altered fecal pH and high mortality, we wished to determine why fecal pH was altered in critically ill patients. We measured fecal organic acids to examine the relation between the fecal organic acids and the fecal pH levels, because abnormally acidic or alkaline pH reflects an abnormality in either acid production or its absorption. In general, intraluminal pH rapidly changes from highly acidic in the stomach to about pH 6.0 in the duodenum, and then gradually increases in the small intestine, which ranges from 5.5 to 7.0. It gradually increases to 6.5 to 7.5 in the distal ileum. In the cecum, the pH decreases to 5.5 to 7.5, but again gradually increases, reaching pH 6.1 to 7.5 in the rectum [5,18]. Bile pH itself can reach 9.0 [19].

In alkaline feces, only propionic acid was significantly lower than that of normal-range feces. Elevated fecal pH suggests inadequate digestion and acid production. H_2 _blockers, which are usually administered to ICU patients, can contribute to decreased acid production. Additionally, administration of antibiotics, which are also used to treat ICU patients, may kill the intestinal commensal microbiota, which leads to reduced production of SCFA. Bacteroidaceae, *Bifidobacterium *spp., and Enterobacteriaceae were significantly decreased in alkaline feces compared with feces from healthy patients. It has been shown that alkaline feces might be undesirable compared with mildly acidic feces [20]. Mildly acidic feces may be preferable because many intestinal pathogens and putrefactive bacteria prefer a neutral pH. SCFA production from prebiotic fermentation and the concomitant decrease in pH may contribute to the reduction of these bacteria [21]. Guimber and colleagues [22] demonstrated that additional a multifibre nutrient mixture with prebiotic components promotes bifidobacteria and reduces stool pH, indicating improved gut health [22]. The mild acidic environment may create a more-favorable environment for the growth of beneficial bacteria as opposed to pathogens and to the stimulation of gut-associated lymphoid tissue.

Although mildly acidic feces can be preferable, severely acidic feces may be problematic. In acidic feces, total organic acids tended to increase. Lactic, succinic, and formic acids were significantly increased in acidic feces. These increased lactic and succinic acids made feces acidic, and they are usually produced by Enterobacteriaceae [23]. This may be because Bacteroidaceae and *Bifidobacterium*, but not Enterobacteriaceae, were significantly decreased in acidic feces compared with those from healthy patients. The presence of low fecal pH might suggest carbohydrate malabsorption, including lactose malabsorption. Because low pH values of feces are often used to diagnose osmotic diarrhea [24], Eherer and colleagues showed that, when diarrhea was caused by carbohydrate malabsorption, the fecal pH was always less than 5.6 and usually less than 5.3 [25]. Moreover, secondary lactose malabsorption can be seen with any form of mucosal injury of the gastrointestinal tract that causes villus flattening or damage to the intestinal epithelium. Severe insults cause small-intestinal injury with loss of the lactase-containing epithelial cells from the tips of the villi. The immature epithelial cells that replace these are often lactase deficient, leading to secondary lactose deficiency and lactose malabsorption [26].

Here, a cascade, once normal flora is altered, decreases the number of beneficial bacteria. This causes fecal pH to increase, which could then allow harmful bacteria to proliferate because of the alkaline environment. Harmful bacteria may then produce lactic and succinic acids, which lower the fecal pH and injure the intestinal epithelium. Finally, the injured intestine does not absorb lactose, which causes the fecal pH to be severely acidic.

Some limitations of this study relate to the fact that the timing of fecal sample acquisition was dependent on the patients. The normal pH values of feces may occur in two situations: when the patient is healthy, or at a transient point during the change from alkaline to acidic pH. Our data demonstrate that a significant impact of fecal pH on mortality and morbidity is found, even if transient cases were included in the normal group.

## Conclusions

We clearly showed that abnormal fecal pH is associated with higher incidence of bacteremia and mortality in critically ill patients. Fecal pH may be used as an assessment tool for the clinical course of critically ill patients.

## Key messages

• Incidence of bacteremia increased with pH 6.6 increase.

• Mortality increased with pH 6.6 increase.

• In acidic feces, lactic and formic acids were significantly higher than those in normal-range feces.

• Bacteroidaceae and *Bifidobacterium *spp. were significantly decreased in both acidic and alkaline feces.

## Abbreviations

CFU: colony-forming unit; MODS: multiple organ failure syndrome; SCFA: short-chain fatty acid; SIRS: systemic inflammatory response syndrome.

## Competing interests

The authors declare that they have no competing interests.

## Authors' contributions

AO, the corresponding author, gathered the data, analyzed them, and wrote the article. KS gathered the data and made a data sheet. HO is the main mentor of AO and revised the manuscript. OT is also a mentor of AO, supporting especially the analysis. TH is a professor in the Department of Biomedical Statistics, who revised all statistics in the article. TA is a chief of Yakult Central Institute for Microbiological Research, who revised especially organic acid and microbiota sections. KN works for Yakult Central Institute for Microbiological Research and measured fecal pH, organic acids, and bacteria. MM works for Yakult Central Institute for Microbiological Research and measured fecal pH, organic acids, and bacteria. YK advised AO on the whole study design and discussions. TS did the final revision. All authors read and approved the final manuscript.
